# An Uncommon Link: Syndrome of Inappropriate Antidiuretic Hormone Secretion (SIADH) in the Setting of Myeloproliferative Neoplasms

**DOI:** 10.7759/cureus.104231

**Published:** 2026-02-25

**Authors:** Sangya Sharma, Aariez Khalid, Marianne E Yassa, Emad Al Jaber

**Affiliations:** 1 Internal Medicine, University of South Alabama, Mobile, USA; 2 Internal Medicine, USA Health University Hospital, Mobile, USA; 3 Pathology, University of South Alabama, Mobile, USA; 4 Nephrology, University of South Alabama College of Medicine, Mobile, USA

**Keywords:** hypo-osmolality, polycythemia vera (pv), rheumatoid arthritis, syndrome of inappropriate secretion of antidiuretic hormone (siadh), systemic hypertension

## Abstract

Syndrome of inappropriate antidiuretic hormone secretion (SIADH) is a well-known cause of hyponatremia, frequently associated with small-cell lung carcinoma. Its association with myeloproliferative neoplasms (MPNs), however, is rare and underreported. We describe the case of an 81-year-old female with a history of chronic hyponatremia attributed to SIADH, requiring multiple hospitalizations. During her hospitalizations, she would be on fluid restriction and urea tablets. Her medical history included paroxysmal atrial fibrillation, a stable pulmonary nodule, and seropositive rheumatoid arthritis. Initial hematologic evaluations for cytopenias were unremarkable. Nearly a decade later, she developed pancytosis, and subsequent testing revealed a JAK2 V617F mutation with a 40.1% clone size. A bone marrow biopsy confirmed a diagnosis of polycythemia vera, an MPN. Treatment with intermittent phlebotomy and ruxolitinib led to hematologic improvement and complete normalization of serum sodium levels without the need for continued SIADH-specific therapy. This case highlights a rare but significant association between MPNs and SIADH. Proposed mechanisms include ectopic antidiuretic hormone (ADH) production by malignant cells and microvascular changes promoting ADH release. The resolution of hyponatremia with MPN-directed therapy suggests a causal relationship. Clinicians should consider MPNs in the differential diagnosis of unexplained SIADH. Further research is warranted to elucidate the underlying pathophysiological link.

## Introduction

Syndrome of inappropriate antidiuretic hormone secretion (SIADH) is a common and well-recognized cause of hyponatremia, characterized by the excessive release of antidiuretic hormone (ADH), leading to water retention and dilutional hyponatremia [1]. Although SIADH is most commonly associated with malignancies such as small-cell lung carcinoma (SCLC) and other solid tumors [2], it is also known to occur in a range of hematologic malignancies, including myeloproliferative neoplasms (MPNs) [3]. MPNs, a group of clonal hematopoietic stem cell disorders, include conditions such as polycythemia vera, essential thrombocythemia, and primary myelofibrosis and are often complicated by various paraneoplastic syndromes, including SIADH [4].

While SIADH has been documented in the context of hematologic malignancies, reports specifically linking SIADH with MPNs are relatively sparse [5]. This makes the case of a patient with polycythemia vera who developed severe hyponatremia with a sodium level below 125 mmol/L noteworthy. In this case, the patient’s hyponatremia was not only severe but also persistent, and it responded positively to the treatment of the underlying polycythemia vera. The association between polycythemia vera and SIADH highlights a potentially underrecognized aspect of MPN pathophysiology, suggesting that these hematologic disorders may contribute to electrolyte imbalances, possibly through mechanisms involving increased erythropoiesis or the release of vasoactive substances [3].

The pathophysiology behind SIADH in MPNs remains poorly understood, but several hypotheses have been proposed. One possibility is that the excessive erythropoiesis and hyperviscosity seen in polycythemia vera may indirectly trigger ADH secretion via mechanisms involving hypoxia or increased intravascular pressure [2]. Alternatively, the inflammatory cytokines and growth factors associated with MPNs could play a role in dysregulating water balance [4]. This case, therefore, underscores the importance of recognizing SIADH in patients with MPNs, particularly when they present with unexplained severe hyponatremia.

Recent literature has emphasized the need for greater awareness of SIADH as a potential complication in hematologic malignancies, given that early detection and management of SIADH can significantly improve patient outcomes [1]. This report adds to the growing body of evidence suggesting that MPNs, though less commonly implicated in SIADH, should be considered as a possible underlying cause of electrolyte disturbances in affected individuals [5].

## Case presentation

Our patient was an 81-year-old woman with a history of chronic hyponatremia secondary to SIADH, requiring multiple hospitalizations, paroxysmal atrial fibrillation on Eliquis, a 0.6 cm lung nodule on chest imaging, and seropositive rheumatoid arthritis on hydroxychloroquine.

She was initially evaluated by hematology/oncology about a decade ago for chronic thrombocytopenia and leukopenia, with an initial normal bone marrow biopsy and a negative multiple myeloma workup.

**Figure 1:**
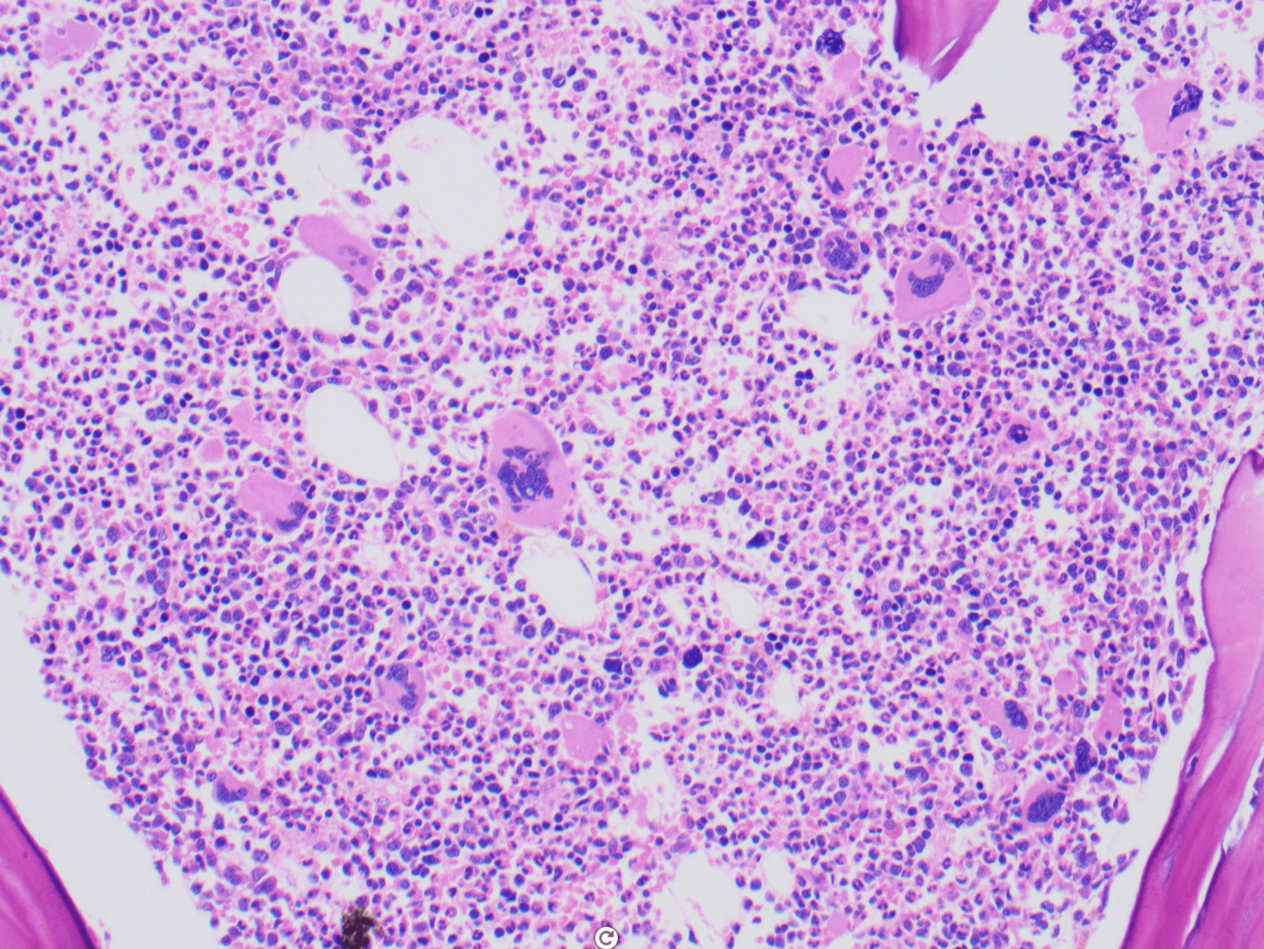
Bone marrow core biopsy showing a hypercellular marrow (80-90%) with panmyelosis (H&E, 20×) H&E: Hematoxylin and eosin stain

This admission, she had pancytosis. Blood smear suggested leukocytosis. Peripheral blood PCR detected JAK2 V617F mutation with a clone size of 40.1%. Bone marrow biopsy revealed hypercellular bone marrow for age, with 90% panmyelosis and abnormal megakaryocytes, consistent with MPN (Figure 1). She was diagnosed with polycythemia vera.

She underwent intermittent phlebotomies with a hematocrit target of <45%. She remained on Eliquis for her paroxysmal atrial fibrillation without complications. She was started on ruxolitinib 15 mg PO for thrombocytosis. Other than pruritus from the polycythemia vera, which improved with monthly dupilumab, she remained asymptomatic. We carefully evaluated potential alternative causes, including her chronic inflammatory state and the stable pulmonary nodule, and found no evidence of acute inflammation, infection, or malignancy progression during the period of hyponatremia.

The diagnosis of SIADH was confirmed by direct measurement of serum osmolality (268 mOsm/kg), which demonstrated true hypotonic hyponatremia, along with inappropriately elevated urine osmolality (249 mOsm/kg), unremarkable urinalysis, stable cortisol level, and natriuresis in the absence of other causes such as volume depletion, renal failure, or diuretic use. These findings were consistent with SIADH and ruled out pseudohyponatremia due to hyperviscosity from polycythemia vera. Additionally, physical exam findings were unremarkable for jugular venous pressure, skin turgor changes, and peripheral edema.

Notably, for hyponatremia, fluid restriction to less than 2 L/day and urea therapy with a goal of serum sodium of 130-135 mmol/L were implemented. Both fluid restriction and urea were discontinued following initiation of MPN-directed therapy. Sodium levels normalized after stopping these interventions, supporting that the correction of hyponatremia was attributable to the MPN therapy (ruxolitinib and phlebotomy) (Table 1).

**Table 1:** Improvement of hyponatremia during hospital course following treatment of MPN The patient initially presented with severe hyponatremia (Na 122 mmol/L on February 23). After the initiation of therapy for the underlying myeloproliferative neoplasm (MPN), the sodium level improved to 130 mmol/L on March 1. On March 10, the level normalized to 135 mmol/L, reflecting the correction of hyponatremia in parallel with the treatment response.

Date	Serum Sodium (mmol/L)	Reference Range (mmol/L)	Status
February 23	122	135–145	Low
March 1	130	135–145	Low
March 10	135	135–145	Normal

## Discussion

The patient had a history of chronic hyponatremia attributed to SIADH, requiring multiple hospitalizations. Despite an extensive diagnostic workup, the underlying cause remained elusive, as no culprit medications, malignancies, pulmonary disease, or intracranial pathology were identified.

SIADH is a common etiology of euvolemic hyponatremia and is classically associated with small-cell lung cancer. However, this was unlikely in our patient, given the extremely slow radiographic progression of her lung nodule. One year later, she was diagnosed with an MPN, prompting reconsideration of whether the hematologic disorder could explain her longstanding SIADH.

Emerging evidence suggests that SIADH in the setting of MPNs may be mediated through several mechanisms. Microcirculatory disturbances, well documented in polycythemia vera, essential thrombocythemia, and myelofibrosis, can create regions of localized tissue hypoxia and metabolic stress. Hypoxia is known to stimulate ADH secretion via activation of hypothalamic osmoreceptors and non-osmotic pathways, providing one plausible mechanism linking MPN-related vascular dysregulation to SIADH [6,7]. Additionally, ectopic ADH production by malignant myeloproliferative cells has been proposed, drawing parallels to the phenomenon described in acute myeloid leukemia (AML), where blast cells were shown to produce ADH and directly induce SIADH [6,8]. Cytokine dysregulation may also contribute; for example, an Asian variant of intravascular large B-cell lymphoma has been reported in which IL-6 secretion triggered inappropriate ADH release, highlighting how hematologic malignancies can alter neuroendocrine pathways [9].

Growing literature supports the concept that inflammatory cytokines, particularly IL-6 and TNF-α, which are elevated in many MPNs, can potentiate non-osmotic ADH release and impair free-water excretion, providing another biologically plausible link between MPN activity and chronic hyponatremia [10]. Furthermore, chronic stress signaling within the bone marrow microenvironment, including aberrant JAK-STAT pathway activation characteristic of MPNs, has been associated with dysregulated neuroendocrine hormone secretion in related hematologic disorders [11]. Although direct evidence connecting MPNs to SIADH remains limited, these mechanistic pathways support a causal framework that is consistent with the clinical trajectory observed in this patient.

## Conclusions

MPNs can contribute to the development of hyponatremia and SIADH, though the exact mechanisms remain unclear. Potential contributing factors may include cytokine dysregulation, paraneoplastic hormone secretion, or altered renal handling of water and electrolytes. Given the rarity of documented cases, this association is likely underrecognized in clinical practice. Early identification and treatment of the underlying hematologic malignancy can lead to a significant improvement in electrolyte abnormalities. In this case, the decade-long interval seen between the initial unremarkable hematologic evaluation and the eventual diagnosis of polycythemia vera emphasizes the importance of longitudinal follow-up in patients with chronic, unexplained electrolyte disturbances. Further research is essential to elucidate the pathophysiological links between MPNs and SIADH and to guide more effective diagnostic and therapeutic approaches in these patients.
